# Food safety knowledge, attitude, and practice of street food vendors and associated factors in low-and middle-income countries: A Systematic review and Meta-analysis

**DOI:** 10.1371/journal.pone.0287996

**Published:** 2023-07-13

**Authors:** Belay Desye, Amensisa Hailu Tesfaye, Chala Daba, Gete Berihun

**Affiliations:** 1 Department of Environmental Health, College of Medicine and Health Sciences, Wollo University, Dessie, Ethiopia; 2 Department of Public Health, College of Medicine and Health Sciences, Adigrat University, Adigrat, Ethiopia; 3 Department of Environmental and Occupational Health and Safety, Institute of Public Health, College of Medicine and Health Sciences, University of Gondar, Gondar, Ethiopia; Wachemo University, ETHIOPIA

## Abstract

Access to safe food is considered a basic human right, but food-borne disease presents a significant public health concern globally. The problem is exacerbated in low- and middle-income countries. Due to the rise in urbanization and the popularity of street food in low- and middle-income countries, understanding the Knowledge, Attitude, and Practice (KAP) of street food vendors is crucial to ensuring food safety. Therefore, this review was aimed to estimate the pooled proportion of KAP of street food vendors toward food safety and its associated factors in low- and middle-income countries. A comprehensive search of published studies before January 30, 2023, was identified using databases like PubMed/MEDLINE, Cochrane Library, HINARI, Science Direct, and African Journals Online, as well as other sources. The preferred reporting items for systematic reviews and meta-analysis guidelines were followed. Data were extracted using Microsoft Excel, and analysis was performed using STATA 14/SE software. The quality of the included studies was assessed using the Joanna Briggs Institute’s quality appraisal tool. A random-effects model was used to estimate the pooled proportion of KAP of street food vendors toward food safety and associated factors. The funnel plot and Egger’s regression test were used to assess publication bias, and I^*2*^ test statistics were used to assess heterogeneity. Furthermore, sensitivity analysis and subgroup analysis was also conducted. In this study, fourteen eligible studies with a total of 2,989 study populations were included. The pooled proportions of good knowledge 62% (95% CI: 51–73), positive attitude 66% (95% CI: 47–86), and good practice 51% (95% CI: 36–65) toward food safety were found among street food vendors. Being of secondary school education (OR = 5.95, 95% CI: 4.05–7.85), having training in food safety (OR = 4.64, 95% CI: 2.62–6.67), having a higher monthly income (OR = 2.98, 95% CI: 1.06–4.9), and having good knowledge of food handling (OR = 2.26, 95% CI: 1.17–3.16) were found to be associated factors in the food safety practice of street food vendors. Based on the findings of this study, there was a significant gap in the KAP of street food vendors toward food safety. Therefore, the provision of training and strengthening health education about food safety are invaluable strategies for improving food safety.

## Introduction

Street foods are ready-to-eat foods and beverages prepared and sold by vendors in public places and on the streets [1]. Millions of people daily feed on street foods, a wide variety of which are easily accessible and relatively cheap [2]. Street food vending businesses contribute significantly to income generation for many individuals from low-income households [3]. Street-vended foods support the dietary diversity of most people in the informal sector because they provide easy access [4, 5]. Street food vending is mostly not regulated by any relevant authority and is often informal [6]. Street-vended foods are often prepared and sold under unhygienic conditions with a lack of basic food service infrastructure and equipment. Due to the lack of these basic services, street-vended foods are considered a food safety hazard for consumers [5, 7, 8].

Access to sufficient amounts of safe and nutritious food is a basic human right and essential to sustaining life and promoting good health [1, 9]. Food safety is one of the integral parts of the Sustainable Development Goals (SDGs) [1]. Food handlers play a major role in ensuring food safety throughout the supply chain (producing, processing, storing, and preparing foods). Food-Borne Diseases (FBD) are major public health concerns around the world, and the problem is exacerbated in low- and middle-income countries due to widespread poor food handling, and sanitation practices, lack of food handler education, lack of food safety awareness, and weak regulatory systems [10–12]. The rising concern about FBD has questioned the levels of KAP of street food vendors [13].

According to the World Health Organization (WHO) report from 2015, the global burden of FBD is estimated at more than 600 million cases, or almost 1 in 10 people, with foodborne illnesses occurring every year and 420,000 deaths. Children under five years of age carry 40% of the FBD burden, with 125, 000 deaths every year, with the highest burden in low- and middle-income countries [14]. The FBD is also responsible for a significant increase in economic expenditures and decreasing the Gross Domestic Product (GDP) of a nation [15].

In low- and middle-income countries, a large proportion of ready-to-eat foods are sold on the street without maintaining their hygienic conditions. Studies have shown that street food vendors are not familiar with the WHO’s Five Keys to Safer Food for food handlers [16], which include keeping clean, separating raw and cooked food, cooking thoroughly, keeping food at safe temperatures, and using safe water and raw materials [9]. Food safety KAP is important because inadequate knowledge, negative attitudes, and poor sanitation practices by street food vendors can cause significant public health problems with food safety issues [17]. Therefore, the KAP of street food vendors on food safety contributes significantly to the occurrence of FBDs among consumers [18].

Street food vendor KAP on food safety has been studied in some parts of the world. According to a study, 67.3% in Ghana [19] and 49.4% in Ethiopia [20] have a good knowledge of street food vendors on food safety. The attitude level of street food vendors toward food safety was found to be 73.89% in Bangladesh [21] and 58.2% in Ghana [19]. On the other hand, the hygienic practice of street food vendors towards food safety was found to be 53% in Ethiopia [22] and 62.9% in Ghana [19]. Educational status, training, vending experience, and monthly income have all been identified as determinants of street food vendors with food safety [19, 22, 23]. The findings of the KAP level of street food vendors and associated factors were found to be inconsistent and did not have the same statistical significance for determining appropriate interventions.

Based on the search of the database, there is no systematic review and meta-analysis conducted on the food safety KAP of street food vendors and associated factors in low- and middle-income countries. Therefore, there is inconsistency between the existing evidence and a significant gap in accessing a comprehensive document regarding the food safety KAP of street food vendors and associated factors. This review can provide well-organized data on the KAP of food safety for street food vendors and associated factors in low- and middle-income countries. "What is the proportion of KAP of street food vendors toward food safety?" and "What are the factors associated with the hygienic practice of street food vendors toward food safety?" were the research questions for this study. Due to the rise in urbanization and the popularity of street food in low- and middle-income countries, understanding the KAP of street food vendors is crucial to ensuring food safety. The findings of this study could help health authorities and non-governmental organizations (NGOs) such as the Food and Agriculture Organization (FAO) and WHO to develop and implement effective strategies to ensure food safety.

## Methods and analysis

### Study setting, registration, and protocol

In this study, the Preferred Reporting Items for Systematic Reviews and Meta-Analysis (PRISMA) guidelines were used [24] **(S1 Table)**. This review protocol was registered in the International Prospective Register of Systematic Reviews (PROSPERO), the University of York Centre for Reviews and Dissemination (Record ID: CRD 42022372726, November 14^th^, 2022). This study was conducted in low- and middle-income countries, according to the World Bank data.

### Information sources and search strategies

A comprehensive systematic literature search was undertaken using PubMed/MEDLINE, Cochrane Library, Science Direct, HINARI, and African Journal Online (AJOL), which were searched up to January 30, 2022. For the PubMed search, the following key terms were used in combination with the Boolean operators "AND" and "OR". ("Food safety" [MeSH Terms] OR "Food hygiene" [All Fields] OR "Food Sanitation" [All Fields]) AND ("Knowledge" [All Fields] OR "Attitude" [All Fields] OR "Practice" [All Fields]) AND ("Street food vendors" [All Fields] OR "Ready-to-eat foods" [All Fields] OR "Food handlers" [All Fields]) AND ("Associated factors" [All Fields] OR "Determinant factors" [All Fields] OR "Identified factors" [All Fields]) AND "low- and middle-income countries" [All Fields].

In addition to the electronic database search, grey literature was searched using Google Search and Google Scholar. Reference lists (bibliographies) of the included studies were also searched to obtain additional articles.

### Eligibility criteria

#### Inclusion criteria

Articles that met the following criteria were considered for inclusion in this review.

- Population: Street food vendors.
- Outcomes: Articles reported the quantitative outcome of the proportional level of KAP of street food vendors and associated factors.
- Study design: A cross-sectional study.
- Study setting: Studies conducted in low- and middle-income countries.
- Language of published articles: Only full-text articles are written in English.
- Publication issue: Peer-reviewed journal articles published before January 30, 2023.

#### Exclusion criteria

In this study, research articles like qualitative studies, systematic reviews, letters to editors, short communications, and commentaries were excluded. In addition, articles that were not fully accessible after three personal email contacts with the corresponding author and articles that did not indicate the overall proportion of KAP of street food vendors and associated factors were all excluded.

### Operational definitions

#### Food safety knowledge

Street food vendors who scored ≥ 70% were considered to have good knowledge, while those who scored < 70% were considered to have poor knowledge [20, 25].

#### Food safety attitude

Street food vendors who scored ≥ 70% were considered to have a positive attitude, while those who scored < 70% were considered to have a negative attitude [20, 25].

#### Food safety practice

Street food vendors who scored ≥ 70% were considered to have good practice, while those who scored < 70% were considered to have poor practice [20, 25].

### Study selection

Two investigators (BD and CD) independently screened articles by their title, abstract, and full text to identify eligible articles using predetermined inclusion and exclusion criteria. The screened articles were compiled together by two investigators (BD and CD), and the disagreement between authors that arises during data abstraction and selection is solved based on evidence-based discussion and the involvement of the third person (AHT).

### Data extraction and management

**Fig 1:**
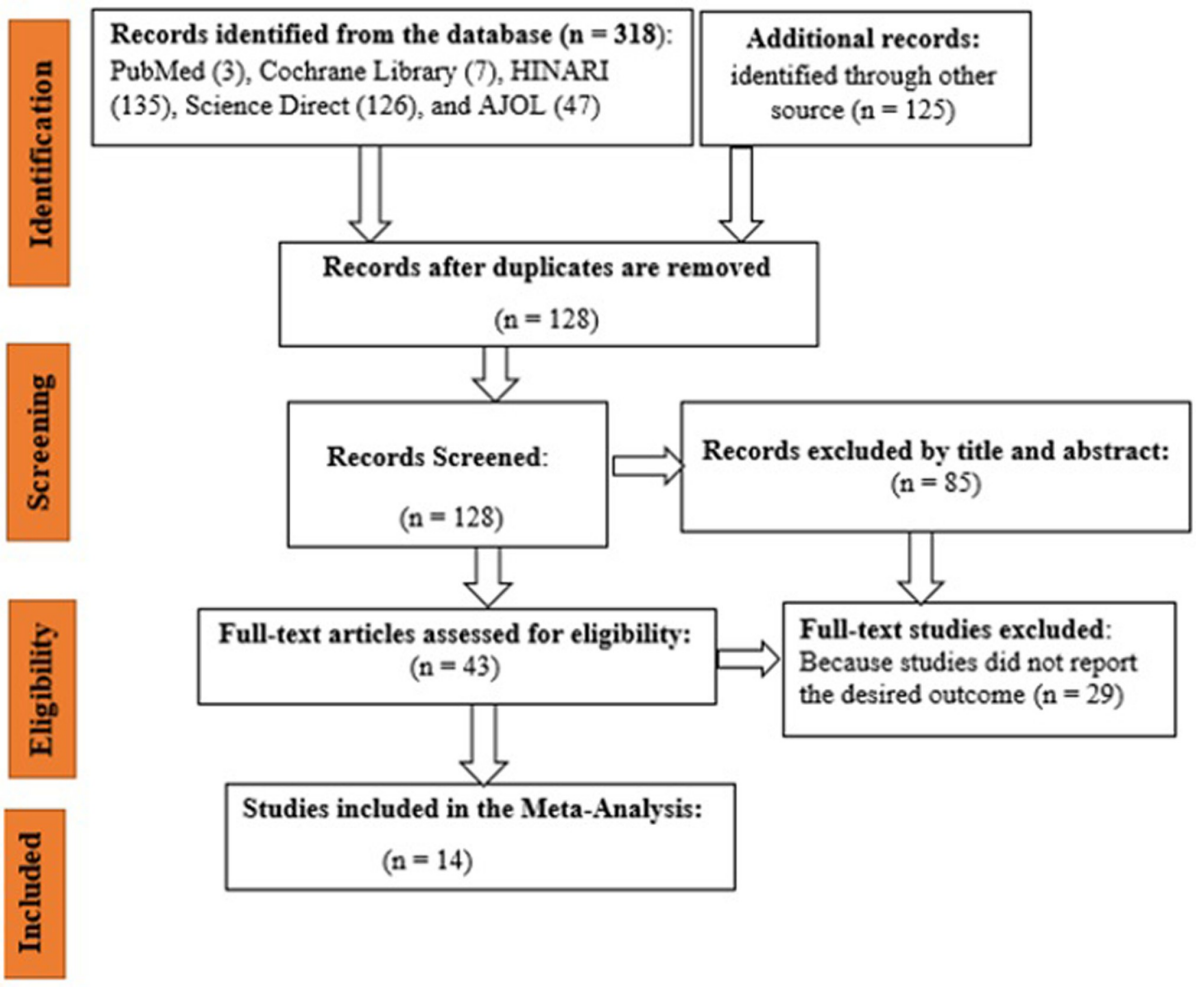
Flow diagram of study selection for this systematic review and meta-analysis, 2023.

The data extraction format was included (name of the author and publication year, study country, method of data collection, sampling methods, sample size, the proportion of KAP of street food vendors, and risk of bias) **(Table 1)**. In this study, to collect and organize search outcomes and removal of duplicate articles, Zotero reference manager software was used. The PRISMA flow diagram was used to summarize the selection process **(Fig 1)**.

**Table 1:** Descriptive summary of included studies in food safety KAP of street food vendors and associated factors in low- and middle-income countries, 2023.

Author, Publication year	Study country	Sampling technique	Methods of data collection	Study design	Sample size	good knowledge (%)	positive attitude (%)	good practice (%)	Risk of Bias
Danikuu et al., 2016 [28]	Ghana	SRS	SQ & OC	CS	150	62.7	-	-	Low
Yahaya et al., 2018 [29]	Nigeria	-	SQ	CS	300	91	93	90.3	Low
Chekol et al., 2021 [23]	Ethiopia	SRS	SQ & OC	CS	422	57.13	-	51.4	Low
Iwu et al., 2017 [30]	Nigeria	CST	SQ & OC	CS	200	81	71	37	Moderate
Jores et al., 2018 [31]	Malaysia	-	SQ & OC	CS	117	22.2	15.4	7.7	Low
Kundu et al., 2021 [32]	Bangladesh	-	SQ	CS	137	-	-	43.1	Moderate
Adane et al., 2018 [22]	Ethiopia	SsRS	SQ & OC	CS	19	-	-	53	Low
Azanaw et al., 2022 [20]	Ethiopia	SRS	SQ	CS	395	49.4	-	49.1	Low
Marutha and Chelule, 2020 [33]	South Africa	SsRS	SQ &OC	CS	312	69.83	-	55.73	Moderate
Meher et al., 2022 [21]	Bangladesh	SRS	SQ	CS	266	71.94	73.89	55.37	Low
Letuka and Nkhebenyane, 2021 [34]	Lesotho	SRS	SQ & OC	CS	48	51	84	84.9	Low
Tuglo et al., 2021 [19]	Ghana	-	SQ	CS	423	67.3	58.2	62.9	Low
Samapundo et al., 2016 [6]	Haiti	-	SQ & OC	CS	80	56	68	40	Moderate
Tesfaye and Tegene, 2020 [25]	Ethiopia	PST	SQ & OC	CS	120	-	-	27.5	Moderate

**Keys:** SQ = Structured Questionnaire, OC = Observational Checklist, SRS = Simple Random Sampling, SsRS = Systematic Random Sampling, CS = Cross-Sectional, CST = Convenient sampling technique, PST = Purposive Sampling Technique, and— = Not found

### Quality assessment of the studies

To assess the quality of the included articles and the risk of bias in each study, the Joanna Briggs Institute (JBI) quality appraisal tools for analytical cross-sectional studies were used [26]. Two reviewers (BD and CD) independently assessed the quality of the included articles. The assessment tool contains eight criteria: (1) clear inclusion and exclusion criteria; (2) description of the study subject and study setting; (3) use of a valid and reliable method to measure the exposure; (4) standard criteria used for measurement of the condition; (5) identification of confounding factors; (6) development of strategies to deal with confounding factors; (7) use of a valid and reliable method to measure the outcomes; and (8) use of appropriate statistical analysis. It was evaluated using the JBI critical appraisal checklist of cross-sectional study options: yes, no, unclear, and not applicable. The risks for biases were classified as low (total score, 6 to 8), moderate (total score, 3 or 5), or high (total score, 0 to 2). Finally, articles with low and moderate biases were considered in this review **(S2 Table)**.

### Outcome of interest

There are two main outcomes of this study. The primary outcome of this study was the pooled proportion of KAP for street food vendors. It was determined using a percentage (%). The pooled measure of association between the hygienic practice of street food vendors and associated factors in low- and middle-income countries was the second outcome of interest to this review. It was determined using the pooled odds ratio (OR) with a 95% confidence interval.

### Statistical methods and data analysis

The extracted data were exported from a Microsoft Excel spreadsheet to STATA version 14 for further analysis. Heterogeneity among the included studies was quantitatively measured by the index of heterogeneity (*I*^*2*^ statistics), in which 25%-50%, 50%-75%, >75% represented low, moderate, and high heterogeneity, respectively [27]. The overall pooled estimate KAP of street food vendors was computed using the metaprop STATA command. A subgroup analysis was conducted by a study country to see the difference in the pooled proportion of KAP of street food vendors. The influence of a single study on the overall pooled estimate was assessed using a sensitivity analysis. Furthermore, the small-study effect was evaluated using the funnel plot test and Egger’s regression test, with a p-value <0.05 as a cutoff point to declare the presence of publication bias. A p-value <0.05 was used to declare the association as statistically significant at a 95% confidence level. The results were presented using graphs, tables, texts, and a forest plot.

## Results

### Searching process

Using the database and manual searching, a total of 443 articles were retrieved. After the duplication was removed, there were 128 articles remaining. Based on their titles and abstracts, 85 articles were excluded. In addition, 29 articles were excluded because they did not report the outcome of interest. Finally, 14 articles were included in this study **(Fig 1)**.

### Characteristics of the included studies

In this review, the publication year, study country, methods of data collection, sampling methods, sample size, and proportion of KAP of street food vendors are all compiled in (**Table 1)**. By design, all included studies were cross-sectional. This study included a total of 2,989 participants [6, 19–23, 25, 28–34]. The included articles were conducted between 2014–2022. The included study sample sizes ranged from 19 to 423. The majority of the included studies were conducted using structured questionnaires and observational checklists. Four studies from Ethiopia [20, 22, 23, 25]; two studies from Ghana [19, 28]; one study from South Africa [33]; two studies from Bangladesh [21, 32]; one study from Malaysia [31]; one study from Lesotho [34], two studies from Nigeria [29, 30] and one study from Haiti [6] were used to estimate the pooled proportion of KAP of street food vendors **(Table 1).**

### Pooled proportion of KAP of street food vendors toward food safety

**Fig 2:**
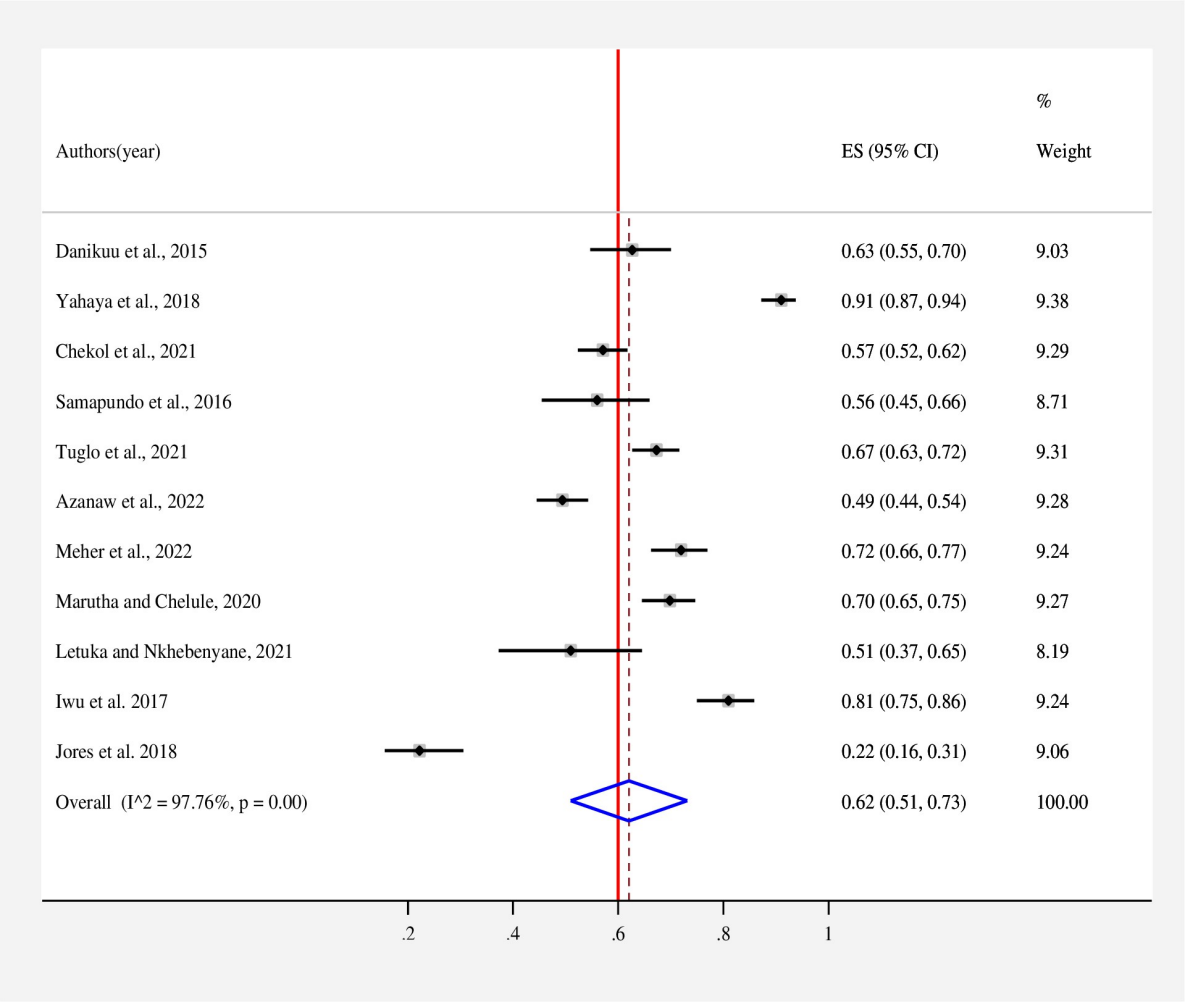
Forest plot for the pooled level of good knowledge of street food vendors on food safety in low- and middle-income countries, 2023. Note: Weights are from random-effects model.

**Fig 3:**
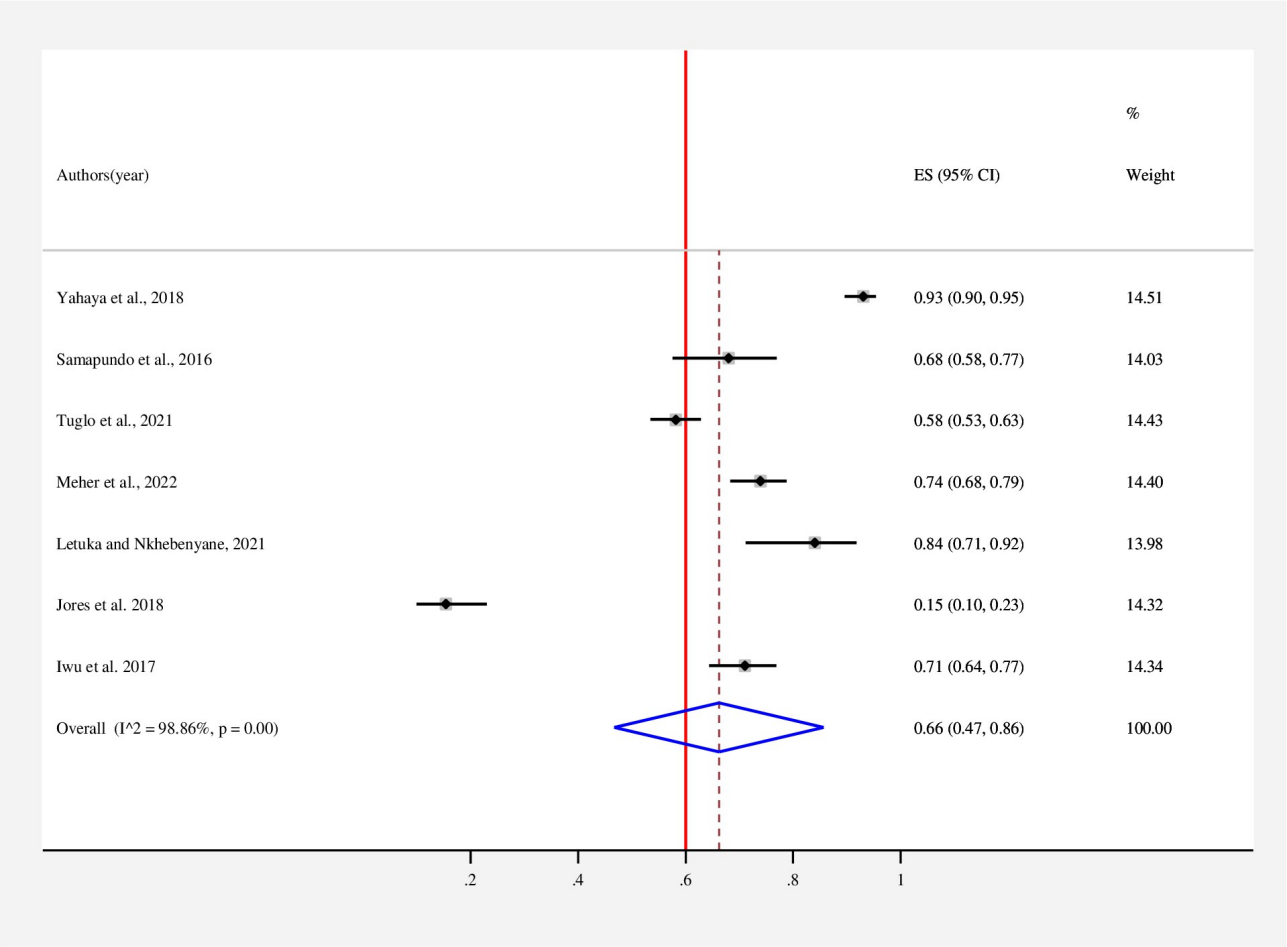
Forest plot for the pooled level of positive attitude of street food vendors on food safety in low- and middle-income countries, 2023. Note: Weights are from random-effects model.

**Fig 4:**
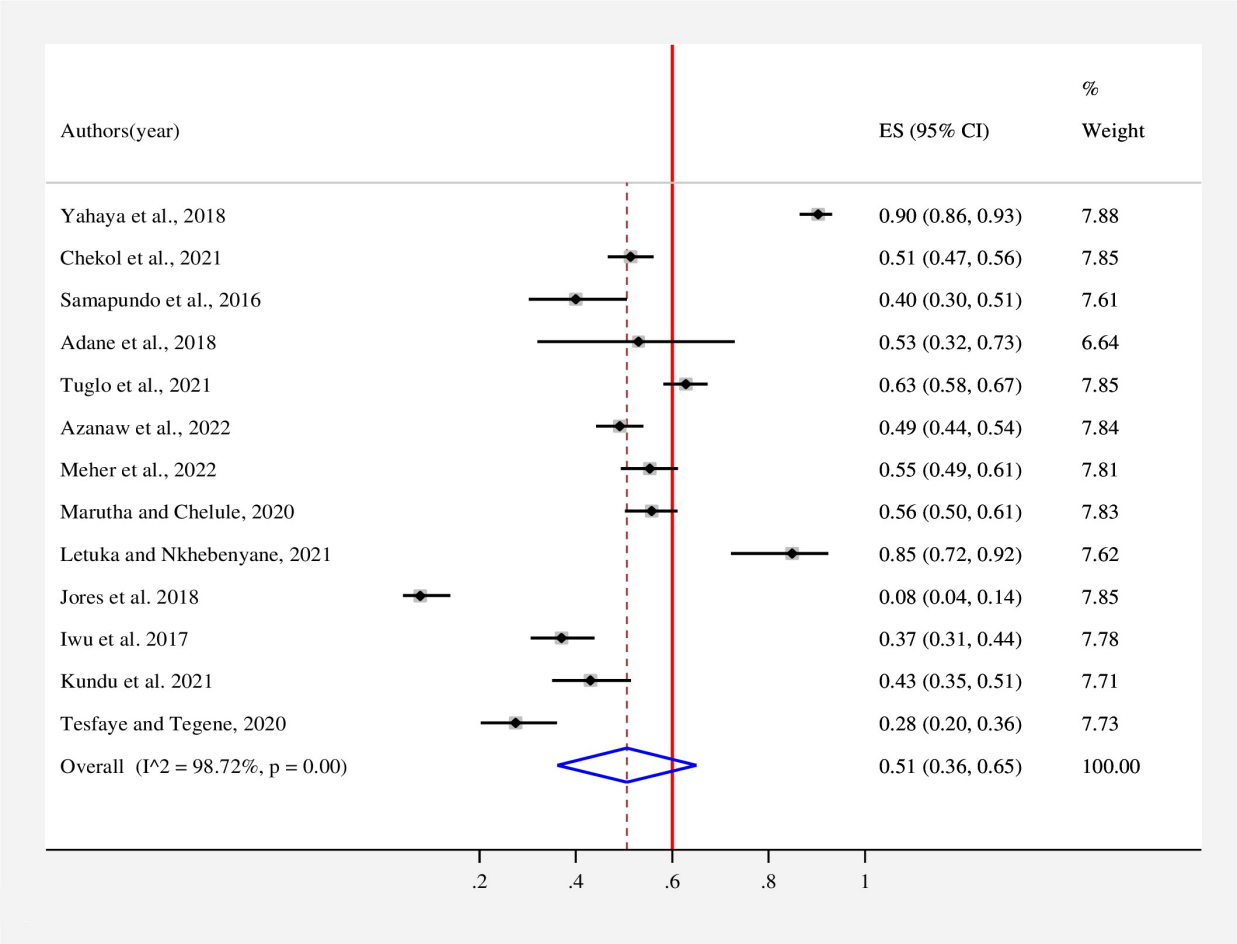
Forest plot for the pooled level of good practice of street food vendors on food safety in low- and middle-income countries, 2023. Note: Weights are from random-effects model.

The estimated overall level of good knowledge **(Fig 2)**, positive attitude **(Fig 3)**, and poor practice **(Fig 4)** of street food vendors toward food safety in low- and middle-income countries is presented in a forest plot. According to the random effects model, the pooled good level of knowledge of street food vendors toward food safety was found to be 62% (95% CI: 51–73; *I*^*2*^ = 97.76%). The pooled estimated level of positive attitude of street food vendors toward food safety was found to be 66% (95% CI: 47–86; *I*^*2*^ = 98.86%). On the other hand, the estimated level of good practice of street food vendors toward food safety was found to be 51% (95% CI: 36–65; *I*^*2*^ = 98.72%).

### Subgroup analysis

In this study, to perform subgroup analysis, a study country was used. As a result, the study’s subgroup analysis of good knowledge level of street food vendors revealed that in Nigeria, 88% (95%CI: 86–91), and Malaysia, 22% (95%CI: 16–31) had the highest and lowest, respectively. Subgroup analysis of the positive attitude of street food vendors found that in Nigeria, 89% (95%CI: 87–92), and Malaysia, 15% (95% CI: 10–23) from the highest to the lowest, respectively. On the other hand, a subgroup analysis of the good practice of street food vendors found that in Lesotho, 85% (95%CI: 72–92), and Malaysia, 8% (95%CI: 4–14), from the highest to the lowest, respectively **(Table 2).**

**Table 2:** Subgroup analysis of pooled levels of good knowledge, positive attitude, and good practices of street food vendors toward food safety in low- and middle-income countries, 2023.

**Knowledge related articles**			
Variables	Characteristics	Included studies	Pooled level of good knowledge
Study country	Ghana	2	66% (95%CI: 62–70)
	Nigeria	2	88% (95%CI: 86–91)
	Ethiopia	2	53% (95%CI: 50–57)
	Haiti	1	56% (95%CI: 45–66)
	Bangladesh	1	72% (95%CI: 66–77)
	South Africa	1	70% (95%CI: 65–75)
	Lesotho	1	51% (95%CI: 37–65)
	Malaysia	1	22% (95%CI: 16–31)
**Attitude related articles**			
Variables	Characteristics	Included studies	Pooled level of positive attitude
Study country	Nigeria	2	89% (95%CI: 87–92)
	Haiti	1	68% (95%CI: 58–77)
	Ghana	1	58% (95%CI: 53–63)
	Bangladesh	1	74% (95%CI: 68–79)
	Lesotho	1	84% (95% CI: 71–92)
	Malaysia	1	15% (95% CI: 10–23)
**Practice related articles**			
Variables	Characteristics	Included studies	Pooled level of good practice
Study country	Nigeria	2	80% (95%CI: 77–83)
	Ethiopia	4	44% (95%CI: 34–55)
	Haiti	1	40% (95%CI: 30–51)
	Ghana	1	63% (95%CI: 58–67)
	Bangladesh	2	51% (95%CI: 46–56)
	South Africa	1	56% (95%CI: 50–61)
	Lesotho	1	85% (95%CI: 72–92)
	Malaysia	1	8% (95%CI: 4–14)

### Heterogeneity and publication bias

**Fig 5:**
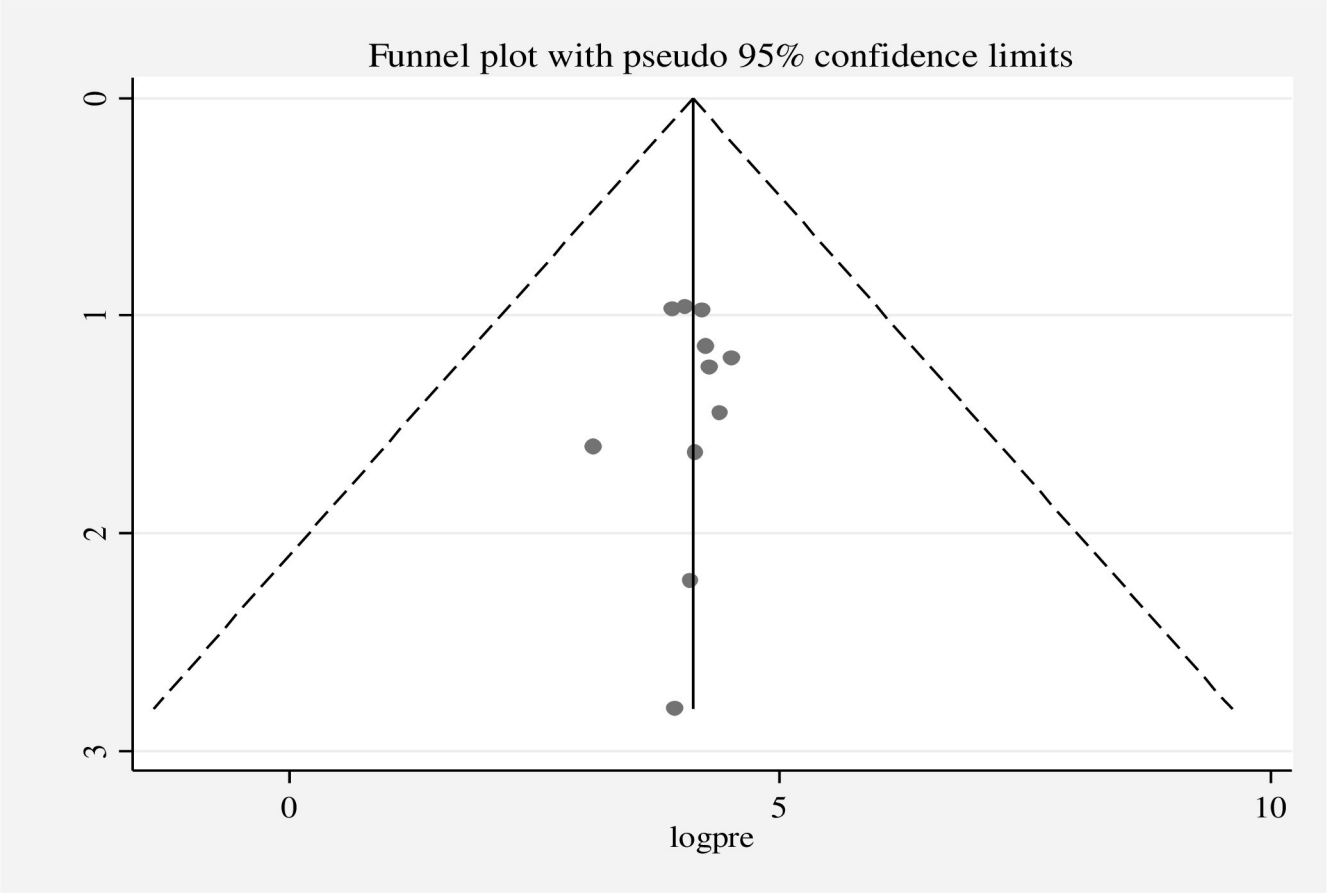
Funnel plot for good knowledge of street food vendors on food safety in low- and middle-income countries, 2023.

**Fig 6:**
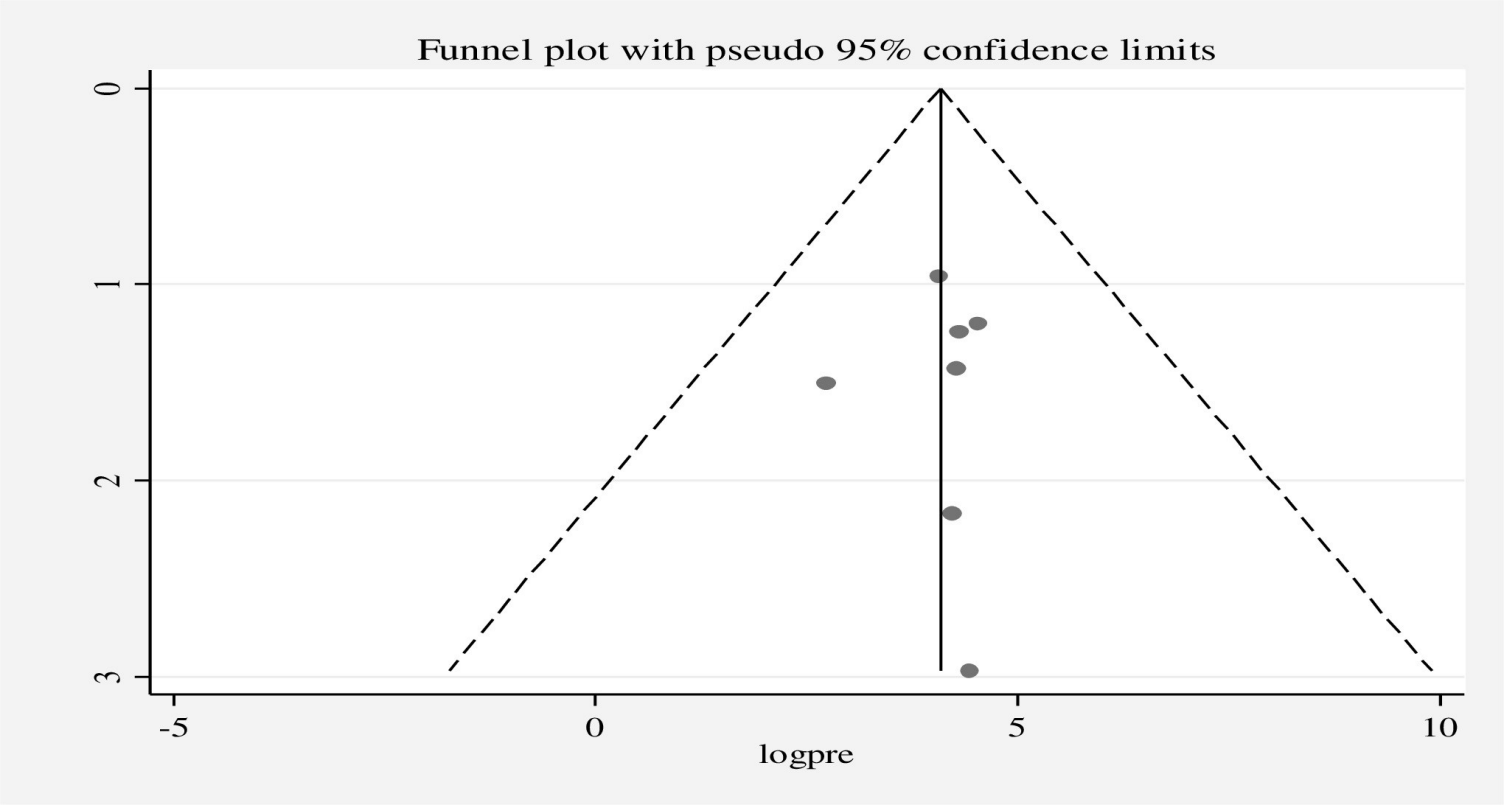
Funnel plot for the positive attitude of street food vendors on food safety in low- and middle-income countries, 2023.

**Fig 7:**
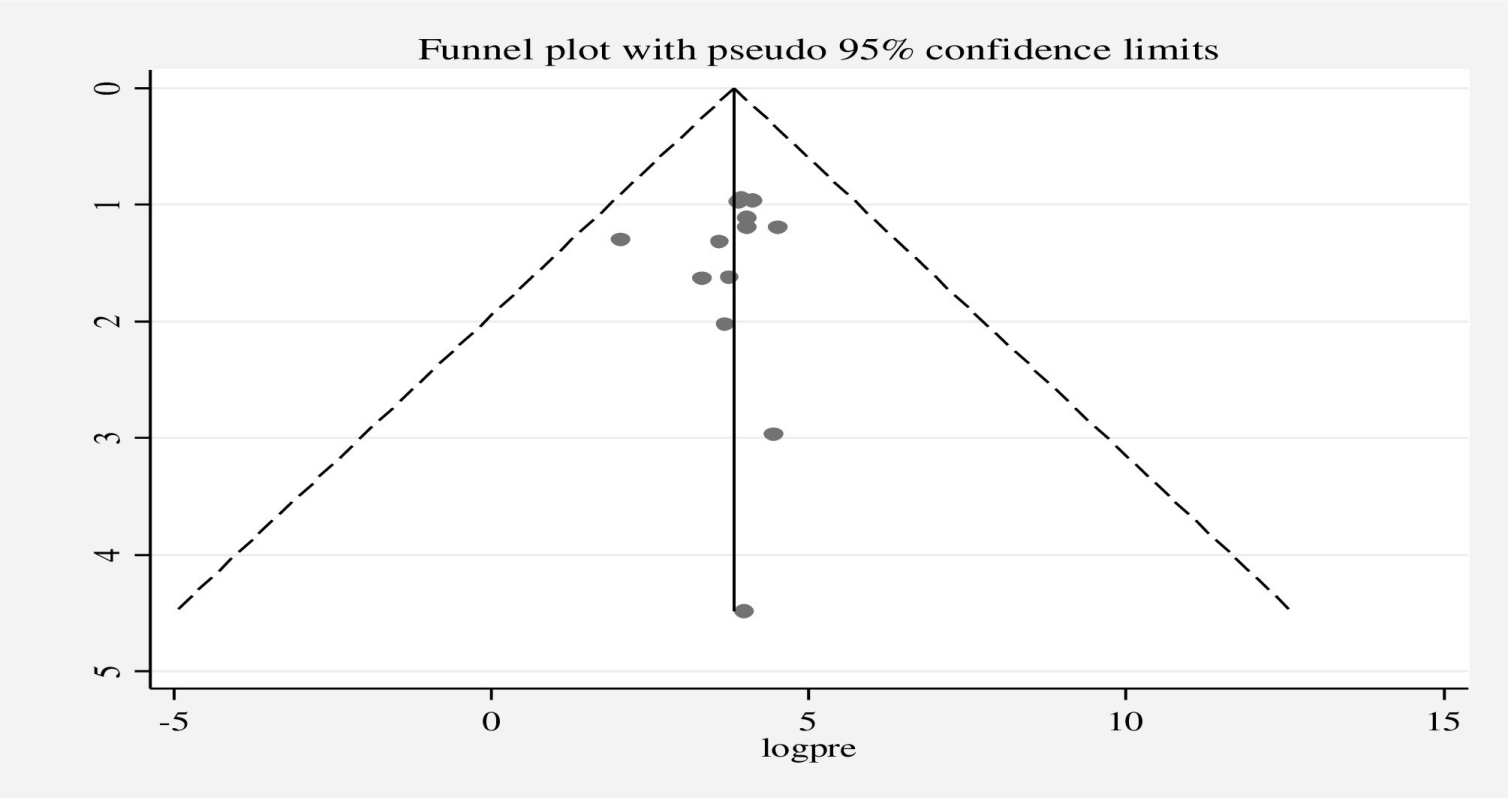
Funnel plot for good practice of street food vendors on food safety in low- and middle-income countries, 2023.

The existence of heterogeneity and publication bias was determined within the included studies. The included studies had a high level of heterogeneity in good knowledge (*I*^2^ = 97.76%, p = 0.00), positive attitude (*I*^2^ = 98.86%, p = 0.00), and good practices (*I*^2^ = 98.72%, p = 0.00). Publication bias was assessed using a funnel plot and the Egger regression test at p-value *<*0.05. A funnel plot for good knowledge **(Fig 5)**, positive attitude **(Fig 6)**, and good practice **(Fig 7)** was found to be some asymmetrical distribution, however, the Egger regression test was found to be not statistically significant for good knowledge (p = 0.058), positive attitude (p = 0.221), and good practice (p = 0.266), respectively. Therefore, the results indicated that there is no strong evidence for the presence of publication bias.

### Sensitivity analysis

A sensitivity analysis was conducted to evaluate the effect of each study on the pooled proportion of good knowledge, positive attitude, and good practice. The results showed that there was no single study effect on the pooled proportion of good knowledge **(S1 Fig),** positive attitude **(S2 Fig)**, and good practice **(S3 Fig).**

### Factors associated with the hygienic practices of street food vendors

**Fig 8:**
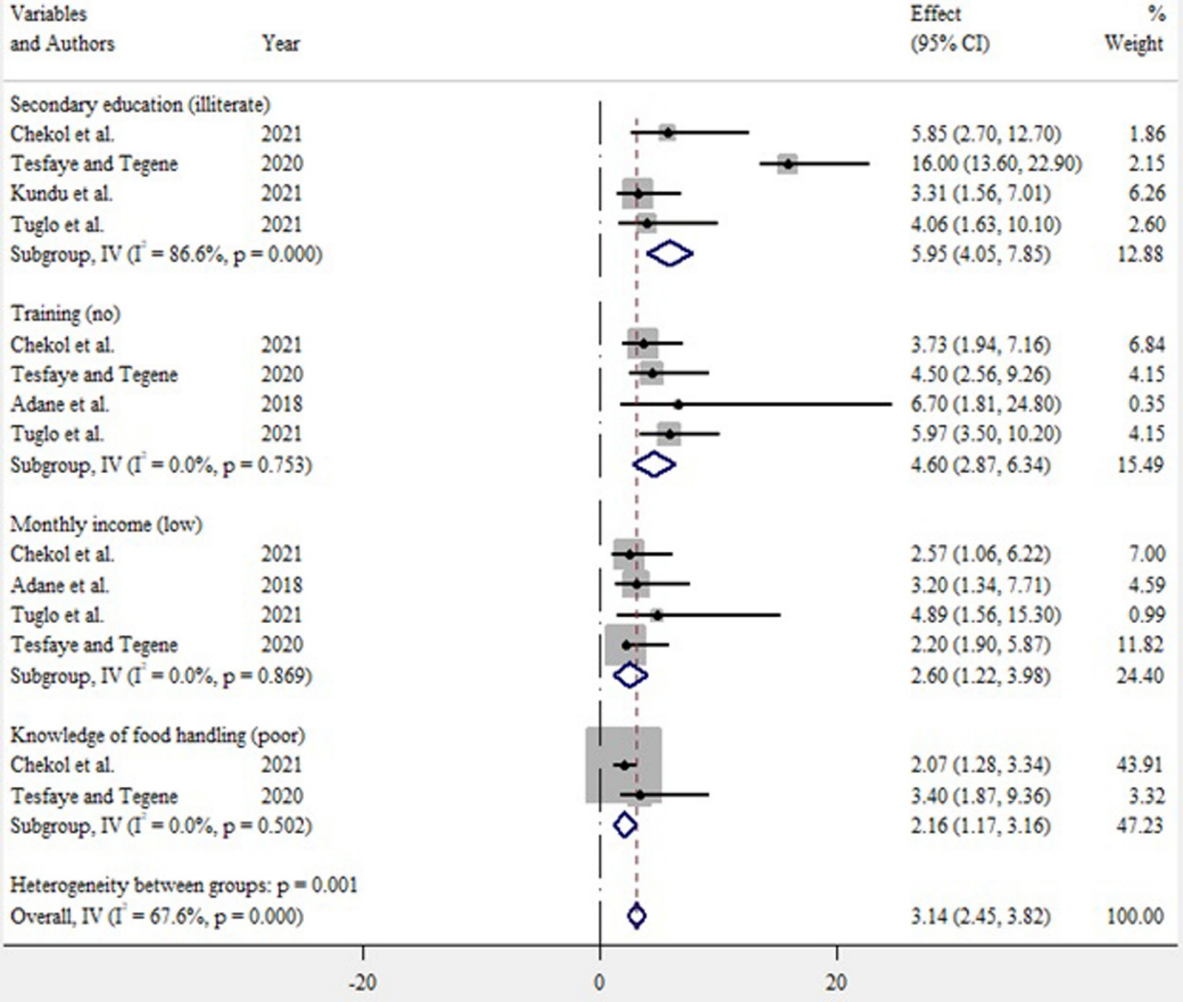
The pooled effect size of factors associated with hygienic practices of street food vendors in low- and middle-income countries, 2023.

In this study, factors associated with the hygienic practice of street food vendors were assessed using 5 studies [19, 22, 23, 25, 32]. Among the 5 studies, the findings of 4 studies [19, 23, 25, 32] revealed that street food vendors with secondary school education were around 6 times more likely to have better hygienic practices than illiterates (OR: 5.95, 95% (4.05–7.85)). In this study, 4 studies [19, 22, 23, 25] revealed that the likelihood of street food vendors having training on food safety were 4.64 times higher to have better hygienic practices than those who did not (OR: 4.64, 95% CI (2.87–6.34)). Four studies [19, 22, 23, 25] revealed that street food vendors with a higher monthly income were 2.60 times more likely to have a better hygienic practice than those with a low income (OR: 2.60, 95% CI (1.22–3.98)). Similarly, 2 studies [23, 25] revealed that street food vendors who have good knowledge of food handling were 2.26 times more likely to have hygienic practices than those who have poor knowledge of food handling (OR: 2.26, 95% CI (1.17–3.16)) **(Fig 8)**.

## Discussion

This systematic review and meta-analysis aimed to determine the pooled proportion of good knowledge, positive attitudes, and good practices of street food vendors on food safety and associated factors in low- and middle-income countries. The safety of street food in low- and middle-income countries was found to be without maintaining their hygienic conditions. Inadequate knowledge, negative attitudes, and poor sanitation practices of street food vendors toward food safety can cause significant public health problems for consumers [13, 17].

According to this study, the pooled proportion of good knowledge of street food vendors toward food safety was found to be 62% (95% CI: 51–73; *I*^*2*^ = 97.76%). This finding is consistent with the study conducted in Ghana [28]. The present finding of good knowledge among street food vendors was found to be lower than a study conducted in Bangladesh (71.94%) [21], Ghana (98.8%) [13], and Ghana (67.3%) [19]. However, it was higher than in a study conducted in Ethiopia (49.4%) [20]. The possible reasons for this discrepancy could be due to variations in the sample size, study setting, and educational level of street food vendors [19]. The knowledge of street food vendors on food safety is crucial to preventing and reducing the spread of foodborne disease, reducing contamination, and decreasing the incidence of diarrhea [20, 21]. Inadequate knowledge of street food vendors is one of the major public health risk factors for the causes of FBD [35]. Therefore, to improve their knowledge and understanding of street food vendors, it is better to take appropriate intervention and provide educational programmes.

The pooled positive attitude of street food vendors toward food safety was found to be 66% (95% CI: 47–86; *I*^*2*^ = 98.86%). This finding is consistent with a study conducted in Haiti [6]. The present finding is lower than a study conducted in Bangladesh (73.89%) [21], Nigeria (93%) [29], and Lesotho (84%) [34]. However, it was higher than in a study conducted in Ghana (58.2%) [19]. The variation might be due to differences in the study population, study settings, and sociodemographic characteristics of street food vendors [19]. The attitude of street food vendors is crucial to understanding the possible ways of food contamination, and it can be significantly improved by training [19, 36].

The pooled good practice of street food vendors toward food safety was found to be 51% (95% CI: 36–65; *I*^*2*^ = 98.72%). This finding is consistent with a study conducted in Bangladesh and Ethiopia [21–23, 37, 38]. The current finding was found to be lower when compared to the studies conducted in Vietnam and Brazil (98.5%) [39, 40] and Ghana (62.9%) [19]. However, the current finding was found to be higher when compared to those in Haiti (40%) [6], Bangladesh (43.1%) [32], Ethiopia (47.14%) [41], and Nigeria (37%) [30]. This discrepancy might be due to differences in knowledge levels among street food vendors, study settings, and socio-demographic factors [23]. Lack of good food safety practice among street food vendors may cause public health problems. Hence, to improve the good practices of street food vendors and to protect the public from different FBD, it is recommended to strengthen health education and intensive training for street food vendors [35].

In this study, a subgroup analysis was conducted by the country to determine whether there is a variation in the KAP levels of street food vendors toward food safety. The findings of good knowledge, positive attitude, and good practice among street food vendors were found to vary from country to country and even within a country. The variation might be due to differences in the study setting and sociodemographic characteristics of the participants [22, 23].

This study also aimed to identify factors associated with the hygienic practices of street food vendors regarding food safety. Accordingly, in this study, education level, training in food safety, income status, and knowledge of food handling were all found to be associated with the hygienic practices of street food vendors towards food safety.

In the current study, street food vendors who have a secondary education level were more likely to have a hygienic practice compared to those who have no formal education. This finding is supported by a study conducted in Ethiopia and Ghana [19, 23, 25, 32, 42]. The potential justification for this might be due to the fact that education might help food handlers to obtain better information regarding food safety compared to those who have not received formal education. In addition, educated food handlers will also be able to read additional written messages on food safety from different sources of information, such as posters and leaflets, which in turn could increase their knowledge of food safety. However, only formal education is not required to ensure food safety; it can also be learned from friends, parents, relatives, and the media about food preparation, food processing, personal hygiene, and the cleanliness of the environment [43, 44].

This study also showed that street food vendors who have received training on food safety are more likely to have good hygienic practices than those who have not. This finding is supported by studies conducted in Ghana and Ethiopia, which suggested that the provisions of training positively influence the hygienic practices of street food vendors [19, 22, 23, 38]. The possible reason might be that street food vendors who have received training on food safety can gain the basic information, experience, and knowledge necessary to maintain foods hygienically because they may receive professional advice during training. This finding assured that the provision of adequate training to food handlers could improve their understanding and hygienic practice. This indicates that providing food safety training to food handlers is invaluable to keeping consumers safe from food poisoning and other related infections that could arise from eating contaminated foods [19, 20, 22].

Similarly, the present study showed that street food vendors with good knowledge of food handling have better hygienic practices than those with poor knowledge of food handling. This finding is supported by a study conducted in Ethiopia, Vietnam, and Saudi Arabia [23, 25, 45, 46]. A good knowledge of food handling practices can be adapted through training. According to the findings, food handlers are better to have good knowledge to prevent foodborne illness [35]. While good knowledge is not a guarantee for good practice, it is one of the main enabling factors for better food handling practices [25].

This study also showed that street food vendors who have a higher monthly income were more likely to have a hygienic practice toward food safety compared to those who have a lower monthly income. This finding is supported by a study conducted in Jordan, Ghana, and Ethiopia that found food handlers with a higher monthly income were more likely to have a hygienic practice compared to those with a lower monthly income [19, 44, 47]. The possible justification for this is that street food vendors with high monthly incomes can afford the hygiene materials needed to maintain their hygienic level and prevent contamination [19, 22, 23].

### Strengths and limitations of this study

This study was conducted using a compressive search strategy using different databases and other sources. In addition, the quality of the included articles was assessed using JBI quality appraisal tools. However, this study considered articles conducted using a cross-sectional design. Hence, a cross-sectional study could not establish cause-and-effect relationships. Furthermore, this study used only articles published in English, which may also limit the conclusiveness of the findings.

## Conclusions

The findings of this study showed that there was a significant gap in the knowledge, attitude, and practice of street food vendors toward food safety in low- and middle-income countries. Lack of formal education, lack of training in food safety, low monthly income, and poor knowledge of food handling were factors that could affect the hygienic practices of street food vendors toward food safety. Based on the findings of this study, continuous training and strengthening health education about food safety, and providing hygienic materials for vendors are invaluable strategies for maintaining and improving the safety of food in low- and middle-income countries. Therefore, the concerned bodies, like NGOs and local governments, should work together to ensure effective implementation of food safety measures among street food vendors. Moreover, future researchers are recommended to incorporate articles published with different study designs and different languages to obtain conclusive evidence.

## Supporting information

S1 Table PRISMA checklist. (DOCX)

S2 Table Results of JBI quality assessment. (DOCX)

S1 Fig Sensitivity analysis for knowledge of street food vendors toward food safety in low- and middle-income countries, 2023. (DOCX)

S2 Fig Sensitivity analysis for attitude of street food vendors toward food safety in low- and middle-income countries, 2023. (DOCX)

S3 Fig Sensitivity analysis for practice of street food vendors toward food safety in low- and middle-income countries, 2023. (DOCX)
