# Supplementary material for: Food safety knowledge, attitude, and practice of street food vendors and associated factors in low-and middle-income countries: A Systematic review and Meta-analysis
Source: PLoS One. 2023 Jul 13;18(7):e0287996. doi: 10.1371/journal.pone.0287996 (PMC10343142; doi:10.1371/journal.pone.0287996)
Supplement: S2 Table — (DOCX) [file pone.0287996.s002.docx]

**Results of JBI Quality Assessment**

| Studies | Clear eligibility criteria | Description of study subject and study setting | Valid and reliable method to measure the exposure | Standard criteria used for measurement of the condition | Identification of confounding factors | Develop of strategies to deal with confounding factors | Valid and reliable method to measured outcomes | Appropriate statistical analysis | Total score out of 8 | Level of bias |
| --- | --- | --- | --- | --- | --- | --- | --- | --- | --- | --- |
| Andy et al. | Unclear | No | No | No | N/A | No | No | Yes | 2 | High |
| Htway and Kallawicha | No | Unclear | No | No | N/A | No | Yes | No | 2 | High |
| Danikuu et al. | No | Yes | Yes | Yes | N/A | No | Yes | Yes | 6 | Low |
| Ntow et al. | No | Unclear | Unclear | No | N/A | No | Yes | No | 2 | High |
| Yahaya et al. | Yes | Yes | Unclear | Unclear | N/A | Yes | No | Yes | 5 | Low |
| Hossen et al. | No | No | Yes | No | N/A | No | No | No | 2 | High |
| Chekol et al. | Yes | Yes | Yes | Yes | N/A | Yes | Yes | Yes | 8 | Low |
| Cortese et al. | No | Unclear | Unclear | Unclear | N/A | No | Unclear | No | 1 | High |
| Tesema | No | Unclear | No | No | N/A | No | No | No | 1 | High |
| Kariuki et al. | No | Yes | Unclear | Unclear | N/A | No | Unclear | No | 2 | High |
| Addo-Tham et al. | No | Unclear | No | No | N/A | No | Unclear | Yes | 2 | High |
| Sarkodie et al. | No | No | Unclear | Yes | N/A | No | Unclear | No | 2 | High |
| Chanthapany et al. | Unclear | Yes | No | No | N/A | No | No | No | 2 | High |
| Shaanika et al. | No | No | No | No | N/A | No | No | No | 1 | High |
| Tesfaye and Tegene | No | Yes | No | Yes | N/A | No | Yes | Yes | 5 | Moderate |
| Samapundo et al. | No | Yes | Unclear | Yes | N/A | No | Yes | Yes | 5 | Moderate |
| Azanaw et al. | No | Yes | Yes | Yes | N/A | No | Yes | Yes | 6 | Low |
| Meher et al. | No | Yes | Yes | Yes | N/A | No | Yes | Yes | 6 | Low |
| Nkosi and Tabit | No | Yes | Unclear | No | N/A | No | Unclear | Unclear | 2 | High |
| Abid et al. | No | No | No | Yes | N/A | No | No | No | 2 | High |
| Marutha and Chelule | No | Yes | Unclear | Yes | N/A | No | Yes | Yes | 5 | Moderate |
| Letuka and Nkhebenyane | No | Yes | Yes | Yes | N/A | No | Yes | Yes | 6 | Low |
| Adane et al. | Yes | Yes | Unclear | Yes | N/A | Yes | Yes | Yes | 7 | Low |
| Tuglo et al. | No | Yes | Yes | Yes | N/A | Yes | No | Yes | 6 | Low |
| Iwn et al. | No | Yes | Unclear | Yes | N/A | No | Yes | Yes | 5 | Moderate |
| Jores et al. | No | Yes | Yes | Yes | N/A | No | Yes | Yes | 6 | Low |
| Kundu et al. | No | Yes | Unclear | Yes | N/A | No | Yes | Yes | 5 | Moderate |
