# Supplementary material for: Food safety knowledge, attitude, and practice of street food vendors and associated factors in low-and middle-income countries: A Systematic review and Meta-analysis
Source: PLoS One. 2023 Jul 13;18(7):e0287996. doi: 10.1371/journal.pone.0287996 (PMC10343142; doi:10.1371/journal.pone.0287996)
Supplement: S2 Fig — (DOCX) [file pone.0287996.s004.docx]

Fig 10. Sensitivity analysis for attitude of street food vendors toward food safety in low- and middle-income countries, 2023.
